# Evaluation of experimental design and computational parameter choices affecting analyses of ChIP-seq and RNA-seq data in undomesticated poplar trees

**DOI:** 10.1186/1471-2164-15-S5-S3

**Published:** 2014-07-14

**Authors:** Lijun Liu, Victor Missirian, Matthew Zinkgraf, Andrew Groover, Vladimir Filkov

**Affiliations:** 1 USDA Forest Service, Pacific Southwest Research Station, Davis CA 95618 USA; 2 UC Davis Genome Center, University of California Davis, CA 95618 USA; 3 Department of Computer Science, University of California Davis, CA 95618 USA

**Keywords:** ChIP-seq bioinformatics, RNA-seq bioinformatics, comparative genomics, chromatin immunoprecipitation sequencing, RNA sequencing

## Abstract

**Background:**

One of the great advantages of next generation sequencing is the ability to generate large genomic datasets for virtually all species, including non-model organisms. It should be possible, in turn, to apply advanced computational approaches to these datasets to develop models of biological processes. In a practical sense, working with non-model organisms presents unique challenges. In this paper we discuss some of these challenges for ChIP-seq and RNA-seq experiments using the undomesticated tree species of the genus *Populus*.

**Results:**

We describe specific challenges associated with experimental design in *Populus*, including selection of optimal genotypes for different technical approaches and development of antibodies against *Populus *transcription factors. Execution of the experimental design included the generation and analysis of Chromatin immunoprecipitation-sequencing (ChIP-seq) data for RNA polymerase II and transcription factors involved in wood formation. We discuss criteria for analyzing the resulting datasets, determination of appropriate control sequencing libraries, evaluation of sequencing coverage needs, and optimization of parameters. We also describe the evaluation of ChIP-seq data from *Populus*, and discuss the comparison between ChIP-seq and RNA-seq data and biological interpretations of these comparisons.

**Conclusions:**

These and other "lessons learned" highlight the challenges but also the potential insights to be gained from extending next generation sequencing-supported network analyses to undomesticated non-model species.

## Background

A major goal of biology is to understand the genetic mechanisms underlying the evolution and development of organisms. To that end, comparative and evolutionary genomic studies are increasingly recognized as being fundamental [1-3]. Such studies are now tractable through the extension of next generation sequencing-based tools and analytical approaches to non-model species [4]. For plants, non-model species fill two important niches. First, many of the most intensively studied model plant species are either domesticated (e.g. maize), or do not fully represent the range of biological processes of interest in plant evolution and development (e.g. Arabidopsis does not display perennial habit). Second, model species have not been developed for many key taxonomic groups.

Forest trees present the opportunity to test the extension of next generation sequencing-based tools and associated analytical approaches to non-model plants. Forest trees are largely undomesticated, and show extremes of plant biology not seen in most model species. One conspicuous feature of trees that is largely lacking in other models is secondary growth, the process by which tree stems grow in diameter and produce wood. Secondary growth is supported by a poorly understood meristem, the vascular cambium, which lies between the inner bark and the secondary xylem (wood) of the stem [5]. The cells of the cambium divide to provide daughter cells that differentiate into the bark or wood tissues. The process of secondary growth is tightly regulated transcriptionally but, although the genes expressed during secondary growth have been previously catalogued using microarrays [6], we currently lack an understanding of how genes are regulated or interact to condition the complex phenotypes seen in the woody stems of trees.

Trees of the genus *Populus *enjoy the most complete set of genomic and experimental tools for any forest trees. Full genome sequence is available for *P. trichocarpa *[7], facilitating use of applications that require mapping sequence reads to non-transcribed regions (e.g. ChIP-seq). However, *P. trichocarpa *is difficult to transform, and thus most labs use other *Populus *species that can be transformed at high frequency for experimental studies of gene function. In a practical sense, *Populus *is of importance for forest industry and biofuels production, and includes keystone species that underpin ecosystems across the Northern Hemisphere [8]. Advances in understanding the basic biology of these species could ultimately be translated into applications of ecological and economic significance [9]. They also represent a test-case for extending advanced genomics and computational approaches to other tree and undomesticated plant species.

Recently we initiated a series of experiments designed to elucidate gene expression and gene regulation involved in secondary growth and wood formation in *Populus *using ChIP-seq and RNA-seq as primary data types. To seed these studies, we selected two classes of transcription factors, Class I KNOX and Class III HD ZIPs, which had been previously implicated as playing key roles in regulating the cambium and wood formation [10-13]. Prior analysis of transcript levels from *Populus *mutants mis-expressing these transcription factors showed mis-expression of overlapping genes or genes influencing similar pathways [10-13], suggesting short path lengths among the Class I KNOX and Class III HD ZIPs in the secondary growth transcriptional network. In addition, the plant hormone auxin has been implicated in the function of both these classes of transcription factors [14,15]. While options for ChIP-seq and RNA-seq experimental design and tools for the resulting data analysis abound, many non-trivial decisions had to be made related to the sequencing and parameter choices, for which guidance is scarce or inexistent for non-model plants.

**Figure 1:**
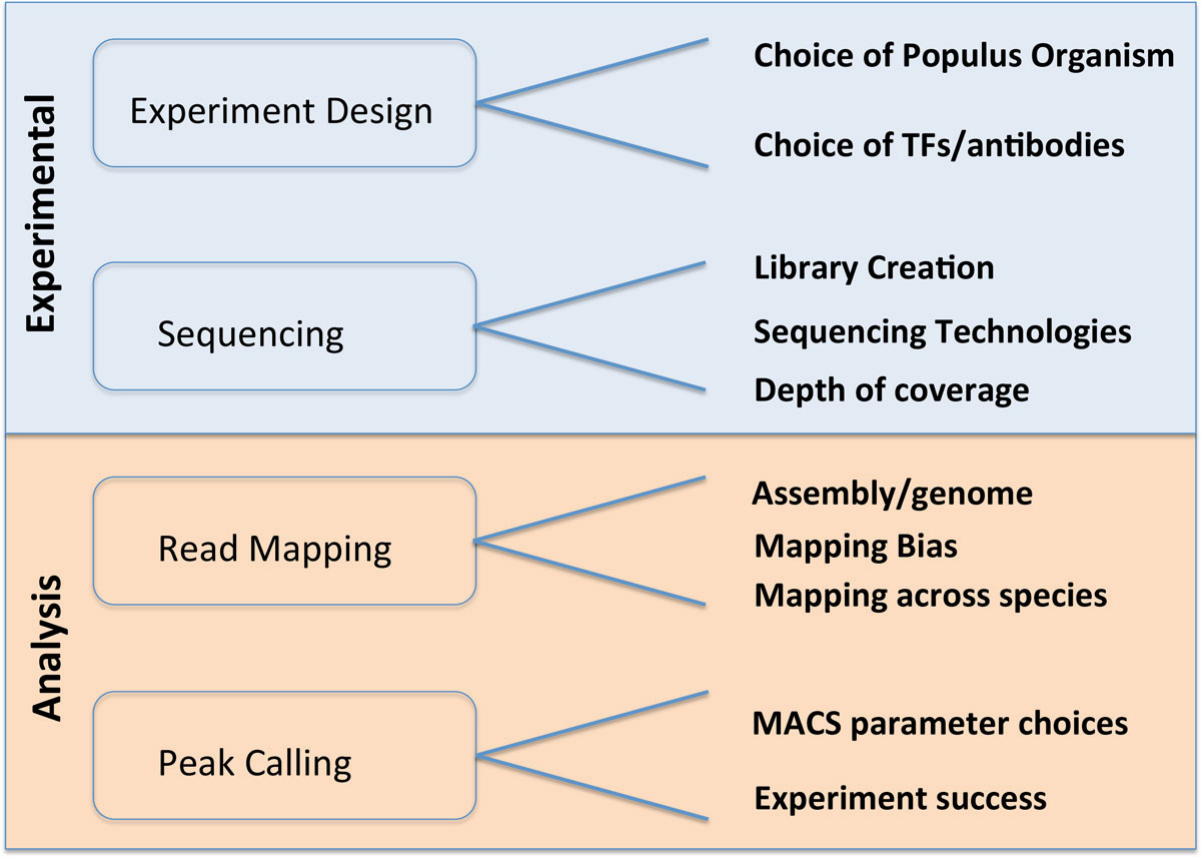
**Overview of the challenges we faced in experimental design and data analysis**.

In this paper, we outline the quantitative and qualitative data analyses that aided us with some of the challenges related to effectively performing gene expression and gene regulation studies in *Populus*, using ChIP-seq and RNA-seq experiments. We faced challenges during all phases of the project, and had to make decisions on a number of practical issues, summarized in Figure 1. Our specific contributions in this paper are the studies that helped us make the appropriate choices for *Populus*. We summarize our results below, and expand on them in the rest of the paper.

• We chose to measure transcript levels using RNA-seq in existing transgenic mutants for Class I KNOX and Class III HD ZIP transcription factors of interest in the *P. tremula *x *P. alba *aspen hybrid background. We performed ChIP-seq experiments in mature *P. trichocarpa*, with at least two peptide-based antibodies raised against each transcription factor based on *P. trichocarpa *gene models.

• We quantify the effects of library creation, choice of sequencing platforms, depth of sequencing coverage, and genome assembly on the quality of short-read mapping, especially cross-species mapping.

• We quantify the effect of different determinants on the downstream ChIP-seq data analyses, including sequence coverage, MACS1.4 parameters, and the use of control sequences.

• We discuss and quantify the effect of multiple samples and replicates as determinants of a successful ChIP-seq experiment.

• We discuss the congruence of ChIP-seq and RNA-seq data and the expectations for genes showing both ChIP-seq peaks and differential expression in mutants.

Because of the generality of the technologies, our results are relevant and can provide guidance not only to those working in *Populus*, but to any emerging model plant organism. Importantly, our studies represent an integrated wet-lab and bioinformatics approach which illustrates both the challenges and promises of next generation sequence-enabled evolution and development studies in non-model species.

## Results and discussion

### Experimental design

Practical issues of the choice of *Populus *as our organism complicated the experimental design. First, transformation is routine in *P. tremula *x *P. alba *aspen hybrid (genotype INRA-717-1B4) but not in the sequenced *P. trichocarpa*. Second, regulations concerning transgenics as well as long generation times prevent growing transformed *Populus *in the field to maturity to harvest large amounts of cambium samples optimal for ChIP. Third, making large numbers of transformants is costly and time consuming. To mitigate these challenges, we chose to measure transcript levels using RNA-seq in existing transgenic mutants for Class I KNOX and Class III HD ZIP transcription factors of interest in the *P. tremula *x *P. alba *aspen hybrid background. We performed ChIP-seq experiments in mature *P. trichocarpa*, to allow harvest of large amounts of ChIP-compatible tissues and to facilitate mapping of short reads to non-coding (and thus less conserved) regions. For the ChIP-seq strategy, at least two peptide-based antibodies were raised against each transcription factor based on *P. trichocarpa *gene models. This strategy allowed ChIP using any *P. trichocarpa *individual, including mature trees, and also facilitated mapping of reads to a reference genome from the same species. In this paper, we will present Class I KNOX ARK1 and RNA PolII ChIP-seq data as examples of TF ChIP-seq. We had two different antibodies for ARK1, designated as ARK1_3738 and ARK1_3940.

### Mapping of short sequencing reads to different genome assemblies

The *P. trichocarpa *genome sequence is currently in its third version, and we designated these different versions as Pt_v1, Pt_v2 and Pt_v3 according to the release date, with Pt_v3 to be the latest version. Each version was produced by different assembly methods, resulting in the highly heterozygous diploid genome being reduced to a single haplotype to various degrees of admixture in the different assemblies. To test whether there were significant differences in mapping efficiencies to different assembly versions, a genomic DNA library, designated as Pt_input, was prepared with genomic DNA from a single *P. trichocarpa *tree and sequenced using Illumina 50 bp single end sequencing (Methods). We multiplexed at most 6 libraries per Illumina lane, which resulted in sequence coverage of 15X-30X depending on the mapping quality outcome, as described below.

The sequencing reads were mapped with Bowtie2.0.2 [16] to each of the three *P.trichocarpa *reference genome assembly versions. As shown in Table 1, there were 47.28%, 47.38%, and 45.02% reads mapped to a single locus (uniquely mapped reads) in Pt_v1, Pt_v2, and Pt_v3, respectively. Additionally, 34.77%, 19.28%, and 28.97% of reads mapped to multiple loci (multiply mapped reads) in Pt_v1, Pt_v2, and Pt_v3, respectively. Thus, while the percentage of uniquely mapping reads is similar among the three versions, different assembly methods lead to significantly different percentages of multiply mapping reads. The percentage of multiply mapping reads directly scales with the total assembled genome size (Table 1), consistent with the idea that the smaller assemblies more aggressively collapse the highly heterozygous diploid genome to a single haplotype, while larger assemblies contain more of the variation present in the diploid genome.

**Table 1:** Mapping Pt_input short sequencing reads to different *P.trichocarpa *genome assembly versions.

Genome reference version	Genome length (Mb)	# of scaffold	% GC	# of genes	# of transcript	Gene model overlap with v3	% of uniquely mapping reads	% of multiply mapping reads
Pt_v3	434.13	1,446	32.88	41,335	73,013	100%	45.02	28.97
Pt_v2	417.14	2,518	32.57	40,668	45,033	82.46%	47.38	19.28
Pt_v1	485.51	22,012	29.68	45,555	45,555	46.93%	47.28	34.77

- Data retrieved from: Pt_v3 - phytozome/v9.1; Pt_v2 - phytozome/v8.0; Pt_v1 - phytozome/v4.1/1.1.

We then compared other aspects of the assemblies. The genome length and GC content are similar across all three assemble versions. However, Pt_v3 has significantly fewer scaffolds (1446) compared to Pt_v2 (2518) and Pt_v1 (22012). Gene annotations are also different across versions, with only 46.93% of the gene models overlapping between Pt_v3 and Pt_v1. Notably, there is also more transcript annotation in Pt_v3 than Pt_v2 and Pt_v1, due to the integration of latest RNA-seq data in Pt_v3 annotation (http://www.phytozome.net/poplar.php). Therefore, despite the slightly lower mapping efficiency, we selected Pt_v3 as the reference assembly of choice because of the lower scaffold number and the superior annotation.

### Challenges of cross-species short-read mapping

While the whole genome reference sequence is only available in *P. trichocarpa *[7], other *Populus *species are routinely used for transformation and experimentation, including the easily transformed *P. tremula *x *P. alba *aspen hybrid utilized by our lab. We tested the efficiency of mapping short-read reads from the aspen hybrid to the *P. trichocarpa *reference genome, to evaluate potential challenges in heterologous mapping.

The percentages of unmapped, uniquely mapped, and multiply mapped reads were determined for genomic DNA libraries, ChIP-seq libraries, and RNA-seq libraries which were subjected to 50 bp single-end Illumina sequencing (Methods). As shown in Table 2, sequencing reads from Pt_input had a significantly higher percentage of uniquely mapping reads (45.04%) than genomic DNA library from hybrid aspen (named as Aspen_input, 29.79%). Similar differences in mapping efficiencies were noted for RNA polymerase II (RNA pol II) ChIP-seq libraries between aspen and *P. trichocarpa *(named as Aspen_RNA pol II and Pt_RNA pol II respectively) (Table 2), which would concentrate the majority of the reads in genic regions that include gene coding, introns, and 5' and 3' untranslated regions. In contrast, the RNA-seq library from aspen (named as Aspen_RNA-seq) showed higher overall efficiency of uniquely mapping reads (51.83%) to the *P. trichocarpa *reference, and actually slightly exceeded the mapping of *P. trichocarpa *RNA-seq library (named as Pt_RNA-seq, 46.51%) in these examples. Additionally, all libraries from *P. trichocarpa *showed higher percentage of multiply mapping reads than the libraries from aspen. As expected, unmapped reads were significantly higher for aspen libraries than *P. trichocarpa *libraries (Table 2). There was no significant difference in average read quality for the Aspen_genomic library versus the Pt_genomic library (data not shown), suggesting that the higher proportion of unmapped reads for the aspen libraries reflects sequence divergence between aspen and *P. trichocarpa*.

**Table 2:** Comparison of short sequencing reads cross species mapping efficiency.

File	% of uniquely mapping reads	% of multiply mapping reads	% of unmapped reads
Pt_input	45.04	28.98	25.98
Aspen_input	29.79	24.41	45.8
Pt_RNA pol II_r1	43.38	13.38	43.24
Pt_RNA pol II_r2	61.69	27.72	10.59
Aspen_RNA pol II	29.44	22.20	48.36
Pt_RNA-seq	46.51	45.60	7.89
Aspen_RNA-seq	51.83	24.82	23.35

To further explore mapping bias of short sequencing reads across the *Populus *genome, we next compared mapping efficiencies between genic and intergenic regions. As shown in Table 3, 28.55% of the Pt_v3 assembly is genic regions and 71.45% is intergenic regions. When mapping Pt_input reads to Pt_v3, 37.56% of uniquely mapped reads were assigned to genic regions, while 62.44% mapped to intergenic regions, indicating an enrichment of reads uniquely mapping to genic regions. On the other hand, 30.21% and 69.79% of multiply mapped reads were assigned to genic and intergenic regions, respectively, which were closer to the percentage of whole genome composition. These observations show that there was, overall, higher mapping coverage of genic regions than intergenic regions in the *Populus *genome, consistent with previous studies from other species [17,18].

**Table 3:** Distribution of short sequencing reads mapping in genic vs. non-genic regions.

	Size (bp)	Size % in whole genome	% of uniquely mapped reads	% of multiply mapped reads
Genic region	123,959,649	28.55	37.56	30.21
Intergenic region	310,175,213	71.45	62.44	69.79
Whole genome	434,134,862	100	100	100

% of uniquely mapped reads was calculated by counting total uniquely mapped reads as 100%.

% of multiply mapped reads was calculated by counting total multiply mapped reads as 100%.

In summary, our data showed that short RNA-seq reads from other *Populus *species can map with reasonable efficiency to the *P. trichocarpa *reference. However, mapping of short reads from ChIP-seq and genomic DNA to intergenic regions is a potential challenge for *Populus *species distantly related to *P. trichocarpa*. These results suggest that robust ChIP-seq analysis requires data acquisition from species with available genome sequence, while heterologous RNA-seq reads can be effectively mapped to a genome reference from a closely related species. These relationships may also hold true for other non-model plants, depending on divergence between the species used for data acquisition and as genome reference.

### Effects of parameters and sequencing coverage for ChIP-seq peak calling using MACS1.4

ChIP-seq peaks represent the putative binding sites of the immunoprecipitated DNA-interacting protein. MACS1.4 is a widely used program for ChIP-seq peak detection, and offers several parameters for optimization [19-21]. Here, we used ChIP-seq datasets from both RNA pol II and ARK1 to test the effect of control file size and p-value cutoff on peak discovery. We also evaluate MACS1.4 peak detection with various sequencing coverage depths by downsampling of the same ChIP-seq libraries.

#### Effects of including control sequences

As shown above, genomic input libraries do not map evenly across the *Populus *genome, indicating that sequences from ChIP-seq libraries would be skewed by similar mapping bias. MACS1.4 provides the option of including a control file that allows estimation of sequencing bias across the genome, which is then used in calculating the likelihood of a ChIP-peak at each genomic region sampled [19]. Therefore, we used the genomic DNA library (input) as a control file, as has been suggested by others [18,22]. The effect of including sequences from a control library was evaluated for two experimental ChIP-seq libraries of RNA pol II (Pt_RNA pol II_r1) and the transcription factor ARK1 (ARK1_3738_r2). Different ratios (0.5:1, 1:1, and 1.5:1) of mapped (Methods) input control to ChIP-seq sequences were presented to MACS1.4 using default parameters except p value 1.00E-07 was used. The ratio of control to experimental reads affected the total number of ChIP peaks called, average peak widths, and the "MACS1.4 score" (a measure of confidence calculated by MACS1.4) for both the Pt_RNA pol II_r1 (Table 4) and ARK1_3738_r2 (Table 5) datasets. There were no clear trends for the number of peaks called or mean peak width, but the 1:1 ratio produced the highest MACS1.4 score in both experiments. To compare the outcomes among the experiments with varied ratios of control to experimental reads, the percentage of overlapping ChIP peaks was compared among the experiments. As shown for Pt_RNA pol II_r1 (Table 6) and ARK1_3738_r2 (Table 7), similar repeatability/overlap was seen for 0.5:1, 1:1, and 1.5:1, while the 0:1 experiment had much lower repeatability/overlap with other ratios tested.

**Table 4:** Effects of varying ratio of input control (C) to RNA pol II ChIP-seq (S) in MACS1.4 ChIP-seq peak calling.

C/S ratio	0 :1	0.5 : 1	1 : 1	1.5 : 1
#reads (S)	13,075,907	13,075,907	13,075,907	13,075,907
#reads (C)	0	6,222,374	12,443,567	18,673,093
#peaks	11474	9322	13350	13292
mean width	933.5906	1106.532	877.0872	881.8897
mean score	320.9228	457.1019	486.15	486.0935

#reads was the total number of filtered reads passed into Bowtie2, "S" was the ChIP-seq sample, and "C" was the input control.

**Table 5:** Effects of varying ratio of input control (C) to ARK1_3738 ChIP-seq (S) in MACS1.4 ChIP-seq peak calling.

C/S ratio	0 :1	0.5 : 1	1 : 1	1.5 : 1
#reads (S)	67,680,143	67,680,143	67,680,143	67,680,143
#reads (C)	0	34,232,969	68,470,396	103,734,004
#peaks	19198	13961	15683	16526
mean width	704.9477	821.5005	794.4457	779.2845
mean score	498.1456	447.5715	535.8703	528.0994

#reads was the total number of filtered reads passed into Bowtie2, "S" was the ChIP-seq sample, and "C" was the input control.

**Table 6:** Overlap of peaks returned in different ratios of input control to RNA pol II ChIP-seq.

C/S ratio	0 :1	0.5 : 1	1 : 1	1.5 : 1
0 : 1	100	80.05	79.21	79.28
0.5 : 1	80.05	100	99.55	99.57
1 : 1	79.21	99.55	100	94.12
1.5 : 1	79.28	99.57	94.12	100

Numbers in the table represent % of peaks in smaller set that overlap with at least one peak in larger set.

**Table 7:** Overlap of peaks returned in different ratios of input control to ARK1_3738 ChIP-seq.

C/S ratio	0 :1	0.5 : 1	1 : 1	1.5 : 1
0 : 1	100	97.93	95.75	95.76
0.5 : 1	97.93	100	99.68	99.79
1 : 1	95.75	99.68	100	98.78
1.5 : 1	95.76	99.79	98.78	100

Numbers in the table represent % of peaks in smaller set that overlap with at least one peak in larger set.

These results suggest advantages to including control sequences in MACS1.4 in terms of MACS1.4 score and repeatability of peaks called, with the 1:1 ratio of control to experimental reads giving slightly better MACS1.4 score than the other ratios tested. We thus used a 1:1 ratio in the following analysis, and this standard should be able to apply to other ChIP-seq data analysis.

#### Effect of MACS1.4 p-value cutoff on ChIP-peak calling

**Figure 2:**
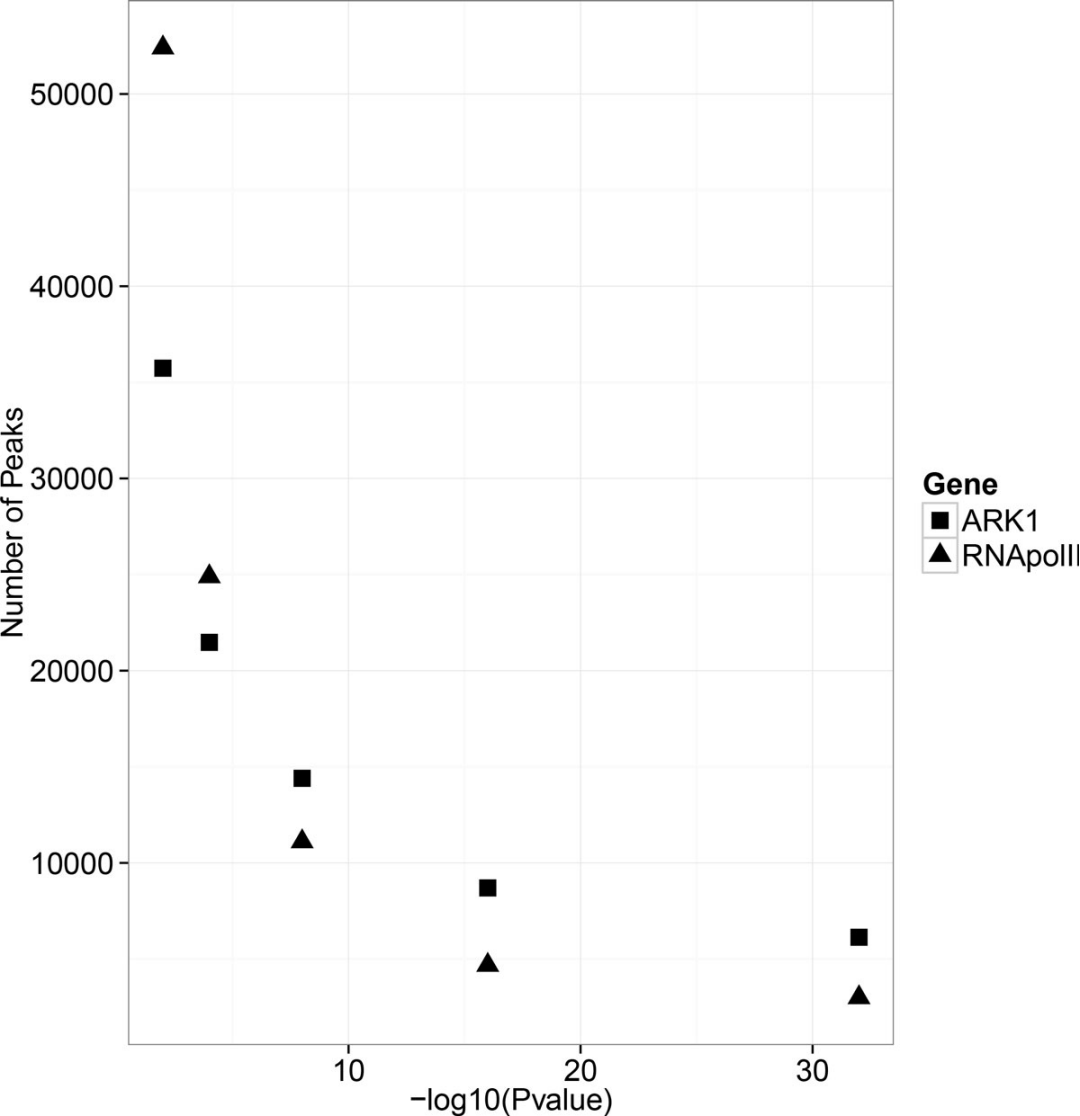
**Effects of p-value on ChIP-seq peaks calling**.

MACS1.4 has an option for selecting a p-value cutoff for ChIP-seq peak calling. MACS1.4 default p-value is 1.00E-05. We tested the effect of different p-values (ranging from 1.00E-02 to 1.00E-32) on the number of peaks called by MACS1.4 for ChIP-seq datasets from RNA pol II (RNA pol II_r1) and ARK1 (ARK1_3738_r2). As shown in Figure 2, there is an inflection point around 1.00E-08 for both the RNA pol II_r1 and ARK1_3738_r2 identifying a reasonable choice for the p-value cutoff. Therefore, we decided to use 1.00E-07 as p-value cutoff for all experiments.

#### Effect of sequence coverage MACS1.4 on ChIP-peak calling

**Figure 3:**
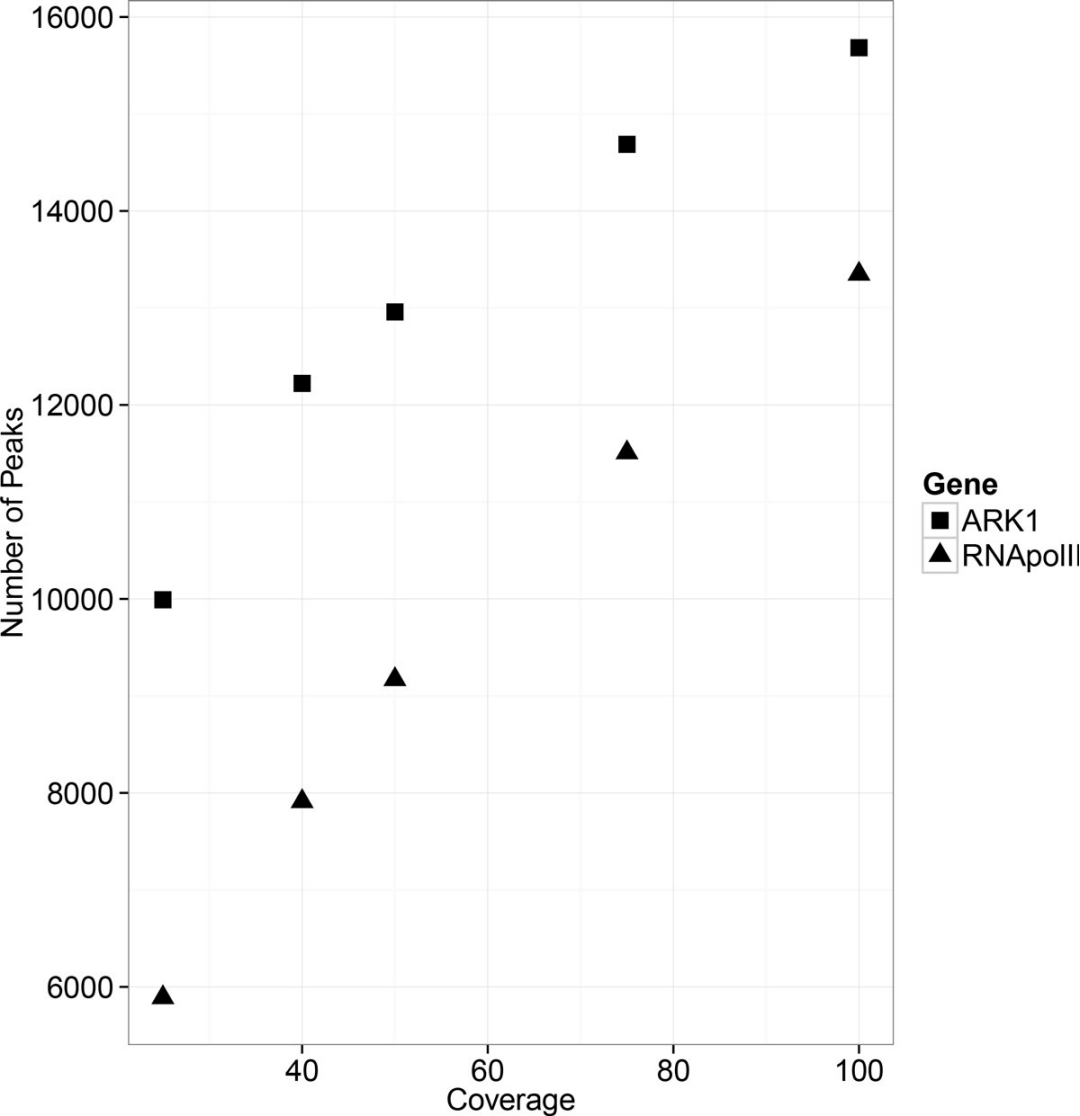
**Effect of sequencing coverage on number of ChIP-seq peaks**. X-axis represents number of detected peaks while Y-axis represents the percentage of reads from the 100% coverage dataset.

**Figure 4:**
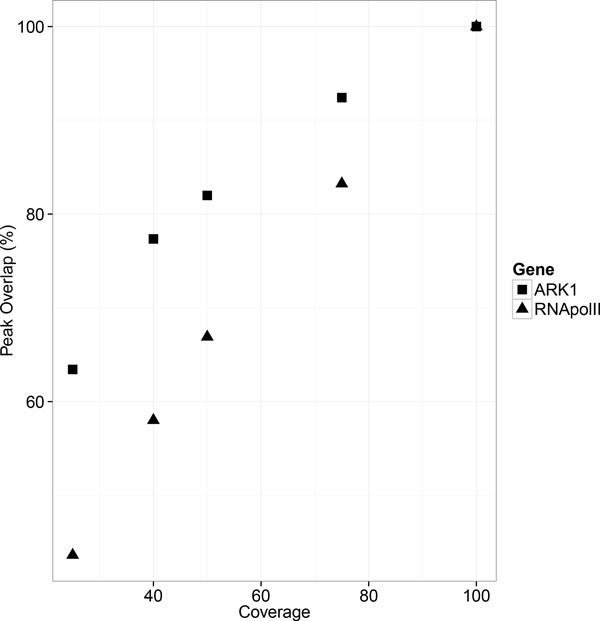
**Effect of sequencing coverage on ChIP-seq peaks robustness**. X-axis represents percentage of sequencing reads while Y-axis represents the percentage of ChIP-seq peaks detected from the 100% coverage dataset.

A critical experimental variable that affects both the cost and outcome of ChIP-seq is the depth of sequencing coverage. Here, the effect of sequencing coverage for peak detection was determined for Pt_RNA pol II_r1 and ARK1_3738_r2 ChIP-seq datasets. Each sample was downsampled to 25%, 40%, 50%, and 75% of the total reads and presented to MACS1.4 with a control file with an equal size for peak calling. As summarized in Table 8 and Table 9, increasing sequencing coverage increased the average MACS1.4 score in Pt_RNA pol II_r1 and ARK1_3738_r2 ChIP-seq experiments, while the mean peak width had maxima occurring between 50-75% coverage in both experiments. As shown in Tables 8, 9 and Figure 3, increasing coverage also increased the number of peaks returned by MACS1.4. To evaluate the robustness of peaks from the different datasets, the percentage of overlapping/repeatable peaks in comparison to peaks detected by the 100% dataset was determined for each analysis, as shown in Figure 4, the percentage of peaks returned increased with increasing sequencing coverage. Together these results indicate that an optimum amount of sequence coverage can't be inferred from these results, and that the optimum coverage in terms of maximizing numbers of peaks and MACS1.4 score lies outside the upper range of sequencing coverage examined in these experiments. This is consistent with the results of tests with human ECODE ChIP-seq datasets, in which they showed that 10 out of 11 DNA-binding factors (typically as transcription factor and their cofactor) cannot reach saturation of peak counts with 30-100 million mapped reads [23].

**Table 8:** Effects of RNA pol II ChIP-seq coverage levels on peak calling.

% of coverage	25%	40%	50%	75%	100%
#reads (S)	3,270,142	5,230,586	6,536,212	9,805,292	13,075,907
#reads (C)	3,111,220	4,976,697	6,222,374	9,332,876	12,443,567
#peaks	5890	7912	9170	11508	13350
mean width (bp)	715.0667	702.0507	773.6713	729.8757	877.0872
mean score	341.7044	381.6625	409.0407	442.2795	486.15

#reads was the total number of filtered reads passed into Bowtie2, "S" was the ChIP-seq sample, and "C" was the input control.

**Table 9:** Effects of ARK1_3738 ChIP-seq coverage levels on peak calling.

% of coverage	25%	40%	50%	75%	100%
#reads (S)	16,922,615	27,069,263	33,843,184	50,761,689	67,680,143
#reads (C)	17,112,375	27,388,564	34,232,969	51,341,520	68,470,396
#peaks	9991	12222	12959	14687	15683
mean width	552.914	686.6993	710.9952	854.1241	794.4457
mean score	349.4032	422.6096	458.1307	503.2893	535.8703

#reads was the total number of filtered reads passed into Bowtie2, "S" was the ChIP-seq sample, and "C" was the input control.

Overall, we found including an equal size of input control file was crucial for ChIP-seq analysis, and the choice of p-value and sequencing coverage affected the ChIP-seq results significantly. These parameters would be expected to have similar impacts for other species.

### Variability of ChIP-seq data across samples and replicates

NGS technologies have fundamentally changed genomic research recently. With cost of sequencing dropping monthly and sequencing capacity likewise increasing, genome level sequencing datasets can be more efficiently generated in short time. On the other hand, NGS also presents challenges in data collection and interpretation. Here we present ChIP-seq datasets from different antibodies and biological replicates to show data variability from raw sequencing reads to the number of MACS1.4 peaks.

We sequenced all ChIP-seq libraries by multiplex sequencing, in which six libraries with specific oligonucleotides (barcodes) were pooled together into a single sequencing lane. Table 10 shows that there were large variations in the number of raw sequencing reads among datasets which was possibly due to unbalanced multiplexing. The raw reads were processed for quality: (1) to trim adaptor contaminations (using the scythe utility at default settings) and (2) to filter out low quality reads (using the sickle utility at default settings) (http://training.bioinformatics.ucdavis.edu/docs/2013/02/bootcamp/galaxy/qa-and-i.html). Most of the sequencing reads passed trimming and filtering (Table 10), and were then mapped onto the *P. trichocarpa *genome, Pt_v3. The fractions of uniquely and multiply mapped reads were similar for all libraries except for the ARK1_3940 antibody, where the mapping was significantly lower. As the libraries of biological replicates of the different antibodies were prepared at the same time, the lower mapping of ARK1_3940 was possibly related to the unique features of this antibody. As discussed in the previous section, sequencing coverage affects the number of MACS1.4 peaks returned, other factors such as antibody binding specificity and peak width might also affect the total number of peaks (Table 10 and 11). For example, in IgG and ARK1_3738 ChIP-seq, the replicates with significantly more reads also got more peaks. On the other hand, in RNA pol II and ARK1_3940 ChIP-seq, replicates with more sequencing reads did not produce more peaks; in this case, the replicate with relatively higher coverage but fewer peaks showed a higher average MACS1.4 score, indicating there were more reads mapped to individual peaks. Also, when comparing sequencing outcomes across antibodies, ChIP-seq runs with similar number of reads may have very different number of MACS1.4 peaks (e.g., the IgG and RNA pol II ChIP-seq replicates). Overall, these results indicate that there are many factors that can introduce variations in ChIP-seq peak calling results, including experimental material, antibody performance, library preparation, and multiplex sequencing. Experiments should be performed with consistent conditions from the Chromatin immunoprecipitation to sequencing.

**Table 10:** Summary of ChIP-seq sequencing datasets.

File	# of raw reads	# of reads after scythe and sickle	% of uniquely mapped reads	% of multiply mapped reads
Pt_Input	114,312,040	103,734,004	45.04	28.98
IgG_r1	9,649,641	9,439,338	41.63	28.70
IgG_r2	29,580,452	28,238,882	52.56	33.13
Pt_RNA pol II_r1	13,436,432	13,075,907	43.38	13.38
Pt_RNA pol II_r2	19,877,305	15,313,223	61.69	27.72
ARK1_3738_r1	25,221,831	22,778,470	42.05	21.69
ARK1_3738_r2	75,397,188	67,680,143	53.61	25.47
ARK1_3940_r1	47,192,418	36,767,421	12.50	11.28
ARK1_3940_r2	30,058,970	27,579,626	36.06	32.72

**Table 11:** Summary of ChIP-seq MACS1.4 peaks.

File	# of MACS1.4 peaks	MACS1.4 score	peaks width (bp)
IgG_r1	197	141.4154	995.4619
IgG_r2	493	122.3166	600.7343
Pt_RNA pol II_r1	13350	486.15	877.0872
Pt_RNA pol II_r2	8688	523.0676	815.1068
ARK1_3738_r1	12155	382.06	555.9356
ARK1_3738_r2	15683	535.8703	794.4457
ARK1_3940_r1	2049	318.0036	513.0278
ARK1_3940_r2	9076	193.6104	348.9233

### Efficacy and quality of ChIP-seq experiments

One of the major challenges when working with ChIP-seq is how to evaluate the results, especially for TFs lacking well-known binding targets. Here, we elaborate on three separate approaches we found useful in evaluating ChIP-seq efficacy and quality.

**Figure 5:**
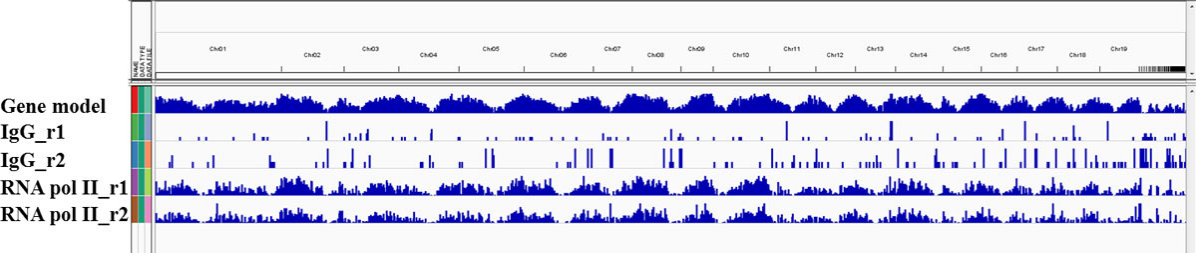
**Overview of IgG and RNA pol II ChIP-seq peaks distribution in *P. trichocarpa *genome using IGV**.

Firstly, RNA pol II and TFs have a function in the regulation of gene transcription, thus, it stands to reason that the majority of their ChIP-seq peaks would be located near genic regions genome-wide [24]. To assess such proximity of binding, we used the Integrated Genome Viewer (IGV) program to visualize genome-wide ChIP-seq binding peaks (Figure 5) [25]. We found that the distribution of IgG ChIP-seq showed no correlation to genic regions. In contrast, the distribution of RNA pol II ChIP-seq peaks was highly coincident with genic regions.

Secondly, there should be significantly higher overlapping of peaks between replicates for specific ChIP-seq binding but not for unspecific binding. As shown in Table 12, there were totally 197 and 493 ChIP-seq peaks, respectively, from the two IgG replicates, and there were only 12 peaks were shared between these two replicates. On the other hand, the peak overlaps between replicates of RNA pol II ChIP-seq was significantly higher: there were 13350 peaks for Pt_RNA pol II_r1 and 8688 RNA pol II _r1 ChIP-seq, and 6499 peaks were overlapping between these two replicates (80% of the peaks from the smaller dataset). High peak overlaps were also detected between ARK1 ChIP-seq replicates: ARK1_3738 had 12155 and 15683 peaks for each of two replicates, with 11075 peaks overlapping between the two (91% of the smaller dataset); ARK1_3940 had 2049 and 9076 peaks for each of two replicates, with 1361 shared peaks between the two (66% of peaks of the smaller dataset). Notably, replicates of ARK1_3940 had lower percentage of overlapping peaks than the ARK1_3738 replicates, indicating less specific binding of the ARK1_3940 antibody.

**Table 12:** Overlap study of ChIP-seq peaks.

File	IgG_r1	IgG_r2	RNA pol II_r1	RNA pol II_r2	ARK1_3738_r1	ARK1_3738_r2	ARK1_3940_r1	ARK1_3940_r2
IgG_r1	197							
IgG_r2	12	493						
Pt_RNA pol II_r1	9	2	13350					
Pt_RNA pol II_r2	16	69	6944	8688				
ARK1_3738_r1	118	39	3210	1610	12155			
ARK1_3738_r2	104	87	4610	2512	11075	15683		
ARK1_3940_r1	152	122	127	168	741	871	2049	
ARK1_3940_r2	157	344	1111	896	3253	3611	1361	9076

Numbers indicate the overlapping peaks between two ChIP-seq datasets.

Thirdly, one should expect low overlap of peaks produced by ChIP-seq of unrelated antibodies. One consideration when designing at least two antibodies for a TF is that if these antibodies recognize the same TF in ChIP-seq, there should be significantly higher overlap between their ChIP-seq peaks than between them and other unrelated antibodies. As shown in Table 12, there was low overlap between ARK1_3738 and IgG ChIP-seq peaks while ARK1_3940 showed higher overlap with IgG, once again indicating that ARK1_3940 had less specific binding than ARK1_3738. However, ARK1_3738 and ARK1_3940 ChIP-seq still showed significantly higher overlap than expected by chance. For example, there were comparable numbers of peaks for ARK1_3738_r1 (12155 peaks) and RNA pol II_r1 (13350 peaks). While 3253 peaks were in the overlap between the ARK1_3738_r1 and ARK1_3940_r2 peaks, there were only 1111 peaks in the overlap between ARK1_3940_r2 and RNA pol II _r1. Similar overlap results were obtained for the other comparisons between ARK1 ChIP-seq and RNA pol II ChIP-seq. These results provide evidence that ARK1_3738 and ARK1_3940 antibodies were targeting the ARK1 protein in our ChIP-seq experiments (albeit the latter may have lower specificity).

Overall, these results suggest that repeatability of peaks generated by different antibodies raised against the same TF is an informative approach for ChIP-seq data quality evaluation.

### Comparing transcriptional activity using RNA pol II ChIP-seq versus transcript abundance with RNA-seq

TFs ChIP-seq binding and RNA-seq expression datasets are regularly combined to identify the direct and indirect targets of regulation [26-28]. However, it is not clear how much correlation should be expected between TF binding to a gene estimated by ChIP-seq peaks and transcript abundance for the gene estimated by RNA-seq, in light of studies that have reported poor correlation for different TFs [29].

**Figure 6:**
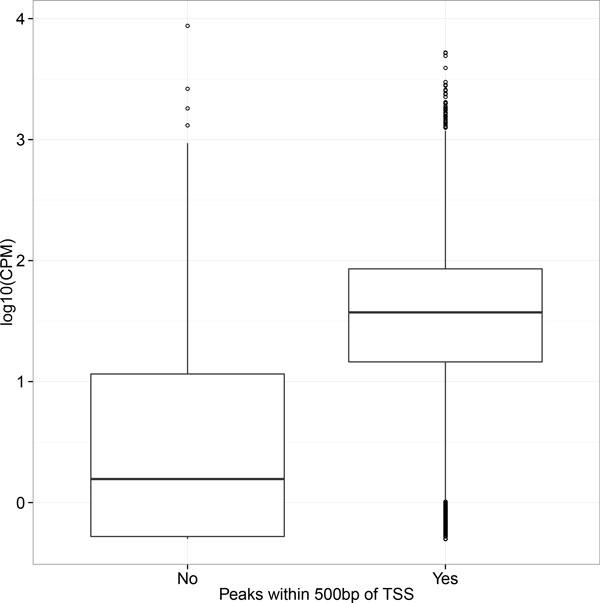
**Gene expression boxplots showing the difference of transcript levels between genes having a Pt_RNA pol II ChIP peak within 500 bp of TSS (right) and genes without such a peak (left)**.

To address this question, we tested the correlation between RNA pol II ChIP-seq binding and the RNA-seq transcript levels from *P.trichocarpa *in vascular cambium tissues. Because RNA pol II is the main RNA polymerase for gene transcription, we hypothesized that RNA pol II ChIP-seq peaks could act as proxy for transcriptional activity. We define genes having peaks within 500bp of the transcriptional start site (TSS) as the target genes of RNA pol II ChIP-seq and used counts per million (CPM) as the measure of transcript levels from RNA-seq. We found that the presence of RNA pol II ChIP-seq peaks had a small but highly significant correlation with transcript abundance measured by RNA-seq (R^2 ^= 0.07, F_1,41333 _= 3254; p-val<2.2x10^-16^), as shown in Figure 6, transcript levels were significantly higher when comparing the population of genes having a peak within 500bp of their TSS to the population of genes without such a peak. This is consistent with previous studies that have shown a weak (R^2 ^< 0.2) correlation between ChIP-seq binding and transcriptional regulation [29]. While RNA pol II ChIP-seq peaks indicates occupancy of RNA pol II at a given locus and RNA-seq detects the final accumulation of transcripts, there could be many other regulation steps between RNA pol II occupancy and transcript levels including transcription activation and transcripts turnover/stability [30,31]. This limited correlation of RNA pol II ChIP-seq binding with RNA-seq transcripts level indicate that in general transcription factor ChIP-seq peaks might have very limited power to identify genes whose transcript levels would be modulated in response to transcription factor binding in RNA-seq.

## Conclusions

We have presented challenges we encountered when generating genome-wide ChIP-seq and RNA-seq datasets from tissues of non-domesticated forest trees of the genus *Populus*. We presented analyses showing the effects of various parameters affecting the outcome of ChIP-seq and RNA-seq analyses, including the differences of mapping to different versions of *Populus *genome assemblies, the challenges of cross-species mapping, the effects of input control file and sequencing coverage for ChIP-seq peak calling, evaluating ChIP-seq data quality technically, and comparisons between ChIP-seq and RNA-seq data. Choices at all these steps could influence the downstream gene expression and gene regulation analyses and results, so they must be approached with care. We hope these findings will be informative for future genomic research in *Populus *and other species.

## Methods

### Plant cultivation and sample collection

Whole stems of *P. tremula *x *P. alba *aspen hybrid hybrid aspen clone INRA 717-IB4 were used for ChIP-seq and RNA-seq experiments from plants that were propagated using previously published methods (Han et al., 2000). Vascular cambium samples were collected during active growth from *P.trichocarpa *trees grown in the field in Westport, Oregon. Briefly, the bark was peeled from stems, vascular cambium and derivatives were collected by light scraping with double edged razor blades. Tissue for RNA-seq was directly frozen in the field in dry ice. For ChIP-seq, tissues were fixed (0.4 M sucrose, 10 mM Tris-HCI, pH 8.0, 1 mM EDTA, 1% formaldehyde and 1 mM PMSF) under vacuum for 15 min. Then, Glycine was added to a final concentration of 0.1 M for 5 min to quench fixation. The tissue was then rinsed in distilled water before being frozen in the field in dry ice.

### Antibody for ChIP-seq

A monoclonal antibody against RNA Polymerase II (MMS-126R) was used for RNA pol II ChIP-seq. ARK1 polyclonal antibodies were produced by Pacific Immunology Corp in rabbits. Briefly, unique and high antigenicity peptides were selected from the ARK1 peptide sequence, synthesized, conjugated, and used in immunizations. Antibodies were purified by affinity columns against the conjugated peptide, and evaluated for titer based on ELISA of the peptide used for immunization.

### ChIP-seq and data analysis

Fixed *P.trichocarpa *vascular cambium or 717-IB4 whole stem samples were ground to powder in liquid nitrogen. The nuclei were isolated with CelLytic PN extraction kit (Sigma-Aldrich) and sonicated in a Lysis buffer (50 mM Tris-HCI, pH 8.0, 10 mM EDTA, 0.5% DOC, 0.3% SDS, proteinase inhibitor and 1 mM PMSF) until the majority of chromatin was fragmented to a size range of 200-500 bp. Chromatin immunoprecipitations were performed using CHIP-IT Express kit (Active Motif) following manufacturer's instruction. Sequencing libraries were prepared with Illumina TrueSeq DNA Sample PrepKit and submitted for ultra-high-throughput Solexa (Illumina) 50 bp single-end sequencing. Input libraries were prepared with approximately 10 ng whole genomic DNA purified from sonicated chromatin used for ChIP reactions.

Sequencing reads were mapped to the *P. trichocarpa *genome using Bowtie2.0.2 (http://bowtie-bio.sourceforge.net/index.shtml) with default parameters. MACS1.4 software (http://liulab.dfci.harvard.edu/MACS/00README.html) was used to call peaks representing enriched binding sites as discussed in the Results and Discussion sections. Size ratio of input control file and ChIP-seq libraries were calculated based on mapped reads of each library.

### RNA-seq and data analysis

*P.trichocarpa *vascular cambium or 717-IB4 whole stem samples were ground to powder in liquid nitrogen. Total RNA were extracted with Trizol (Invitrogen) and then purified with the RNeasy Mini kit (Qiagen) following the manufacturer's protocol. mRNA sequencing libraries were prepared from total RNA using Illumina TrueSeq RNA Sample PrepKit and submitted for ultra-high-throughput Solexa (Illumina) 50 bp single-end sequencing.

Sequencing reads were mapped to the *P. trichocarpa *genome using bowtie2 (For Table 2) or Tophat (http://tophat.cbcb.umd.edu/) (For Figure 6) with default parameters. The raw mapped reads for each sample were counted using htseq-count and read counts per million (CPM) were calculated using the library size normalization function calcNormFactors in the edgeR package [32] (http://www.bioconductor.org/packages/2.13/bioc/html/edgeR.html).

### Data repository

All raw ChIP-seq and RNA-seq data have been deposited in the NCBI SRA database under the accession number SRP028935.

## Competing interests

The authors declare that they have no competing interests.

## Authors' contributions

AG and VF conceived the project. AG and VF managed the project. LL performed the raw data generation. LL, MZ, VF and VM performed the data analyses. LL, AG, VF and VM wrote the manuscript. All authors read and approved the final manuscript.

## Declarations

Funding for publication of this work was provided by USDA AFRI 2011-67013-30062 to AG and VF, and the USDA Forest Service.

This article has been published as part of *BMC Genomics *Volume 15 Supplement 5, 2014: Selected articles from the Third IEEE International Conference on Computational Advances in Bio and Medical Sciences (ICCABS 2013): Genomics. The full contents of the supplement are available online at http://www.biomedcentral.com/bmcgenomics/supplements/15/S5.
